# Blockage of FOXP3 transcription factor dimerization and FOXP3/AML1 interaction inhibits T regulatory cell activity: sequence optimization of a peptide inhibitor

**DOI:** 10.18632/oncotarget.17845

**Published:** 2017-05-13

**Authors:** Teresa Lozano, Marta Gorraiz, Aritz Lasarte-Cía, Marta Ruiz, Obdulia Rabal, Julen Oyarzabal, Sandra Hervás-Stubbs, Diana Llopiz, Pablo Sarobe, Jesús Prieto, Noelia Casares, Juan José Lasarte

**Affiliations:** ^1^ Immunology and Immunotherapy Program, University of Navarra, 31008, IDISNA, Pamplona, Spain; ^2^ Small Molecule Discovery Platform, Molecular Therapeutics Program, Center for Applied Medical Research (CIMA), University of Navarra, 31008, IDISNA, Pamplona, Spain

**Keywords:** Foxp3, Treg, peptide inhibitor, cancer, immunomodulation

## Abstract

Although T regulatory cells (Treg) are essential for the prevention of autoimmune diseases, their immunoregulatory function restrains the induction of immune responses against cancer. Thus, development of inhibitors of FOXP3, a key transcription factor for the immunosuppressive activity of Treg, might give new therapeutic opportunities. In a previous work we identified a peptide (named P60) able to enter into the cells, bind to FOXP3, and impair Treg activity *in vitro* and *in vivo*. Here we show that P60 binds to the intermediate region of FOXP3 and inhibits its homodimerization as well as its interaction with the transcription factor AML1. Alanine-scanning of P60 revealed the relevance of each position on FOXP3 binding, homodimerization, association with AML1 and inhibition of Treg activity. Introduction of alanine at positions 2, 5 and 11 improved the activity of the original P60, whereas alanine mutations at positions 1, 7, 8, 9, 10 and 12 were detrimental. Multiple mutation experiments allowed us to identify peptides with higher FOXP3 binding affinity and stronger biological activity than the original P60. Head to tail macrocyclization of peptide P60-D2A-S5A improved Treg inhibition and enhanced anti-tumor activity of anti-PD1 antibodies in a model of hepatocellular carcinoma. Introduction of a D-aminoacid at position 2 augmented significantly microsomal stability while maintained FOXP3 binding capacity and Treg inhibition *in vitro*. *In vivo*, when combined with the cytotoxic T-cell epitope AH1, it induced protection against CT26 tumor implantation. This study provides important structure–function relationships essential for further drug design to inhibit Treg cells in cancer.

## INTRODUCTION

Regulatory T cells (Treg) play a key role in the homeostasis of the immune system. Their immunosuppressive activity prevents chronic inflammation and maintains peripheral immune tolerance protecting the host against autoimmune diseases [1]. However, this immunoregulatory function restrains the induction of immune responses against cancer and infectious agents [2–4]. Indeed, Treg capable of suppressing the *in vitro* function of tumor-reactive T-cells have been found in humans in many tumors types [5–9] and have been associated with a high death hazard and reduced survival [5, 8].

Treg are characterized by the expression of CD25 and the Treg-specific FOXP3 transcription factor, which is required for their development and function [10–11]. The molecular basis of FOXP3 function has been poorly understood. FOXP3 capacity to bind DNA is critical for its functionality and it is known that FOXP3-DNA interactions are assisted by other cofactors and by multimerization. Indeed, a growing number of transcription factors that interact with FOXP3 are being identified and some have been implicated in the Treg cell–specific gene expression program [12–14]. FOXP3 has various distinguishable functional domains: (i) a N-terminal domain (from a.a. 1 to 193, with two proline-rich regions), (ii) a zinc finger (a.a. 200–223) and a leucine zipper-like motif (a.a. 240–261) (LZ domain) located in the centre of the protein and (iii) the highly conserved carboxy terminal forkhead domain (FKH; from a.a. 338 to 421) responsible for binding to DNA. It has been described that the intermediate region is implicated in FOXP3 dimerization, which is required for its function as a transcriptional regulator [13, 15–17]. Also, physical interaction of this region with the transcription factor AML1 (acute myeloid leukaemia 1)/Runx1 (Runt-related transcription factor 1), suppresses IL-2 and IFN-γ production, upregulates Treg-associated molecules, controls anergy of the cell and exerts Treg suppressive activity [18]. Thus, those strategies able to inhibit FOXP3 dimerization, its interaction with AML1 or to modify the FOXP3 interactome might have important consequences on Treg activity and thus could be exploited as therapeutic agents in cancer.

In a previous work, by using a phage-displayed random peptide library, we identified the 15-mer synthetic peptide P60, which entered the cells, bound to FOXP3 and inhibited murine and human-derived Treg, improving effector T-cell stimulation *in vitro* and *in vivo* [19]. In this work we aimed to identify the region of interaction of P60 with FOXP3, to go in depth on its mechanism of action and to optimize its sequence and improve its activity. We identified the intermediate region of FOXP3 as the region of interaction with P60 peptide and analyzed the impact of this interaction in FOXP3 dimerization and its association with AML1. We have also studied the residues within P60 required for its interaction with FOXP3, and found artificial mutations and modifications that improved its Treg inhibitory potency.

## RESULTS

### P60 binds the intermediate region of FOXP3 and inhibits FOXP3 homodimerization and FOXP3-AML1 heterodimerization

**Figure 1:**
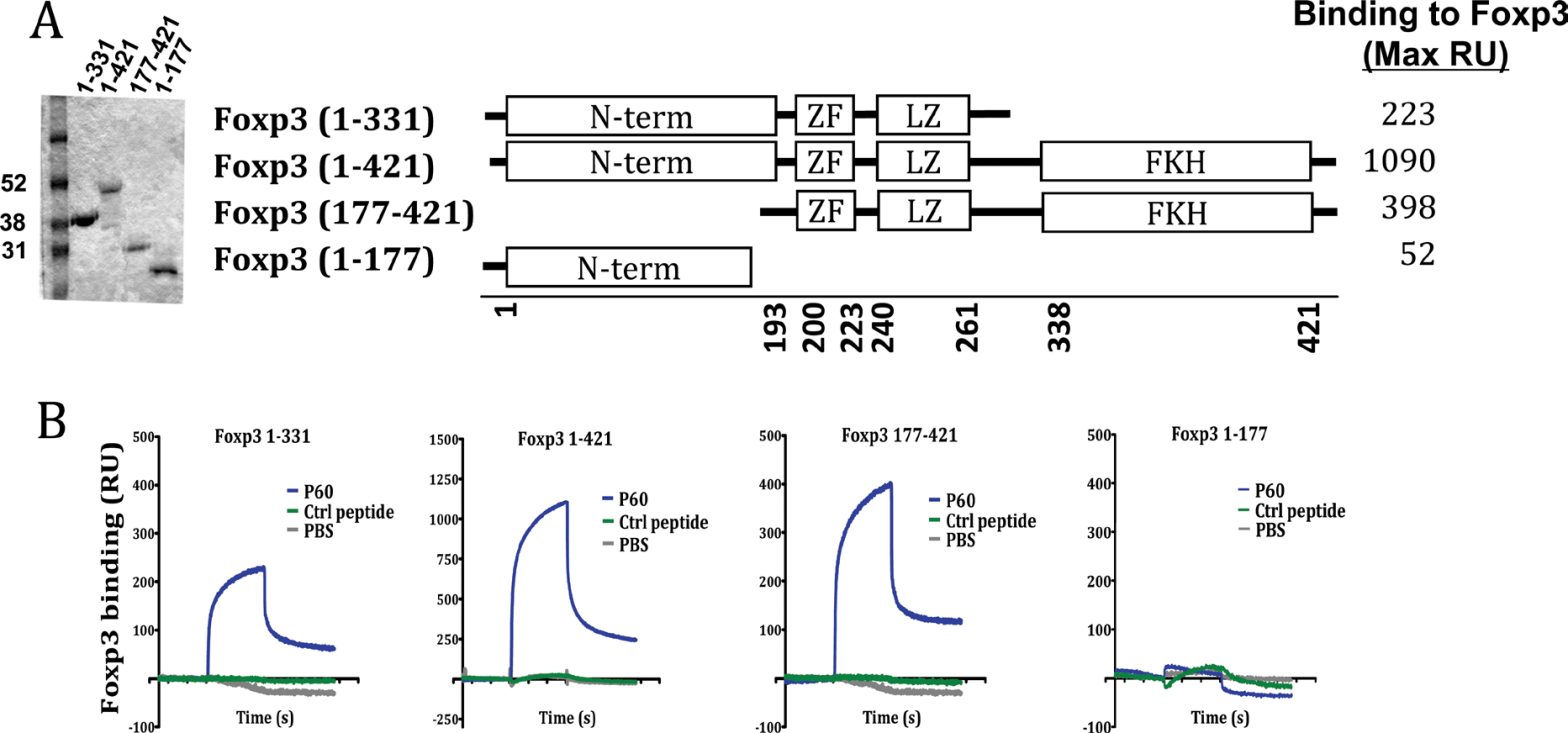
Region of interaction of the FOXP3 inhibitory peptide P60 (**A**) SDS-PAGE analysis of full-length and truncated mutants of FOXP3. Schematic structure of the produced FOXP3 versions and maximal RU of P60 binding to each protein. (**B**) Surface Plasmon resonance sensograms analysing P60 peptide interaction with the indicated protein-coated chip.

Using truncated versions of FOXP3, we attempted to identify the FOXP3 domain interacting with peptide P60, described previously by or group as a FOXP3 inhibitory peptide [19]. Thus, besides native FOXP3 we prepared the deletion mutants FOXP3 (1-331) (lacking the C-terminus of FOXP3), FOXP3 (177-421) (lacking the N-terminus) and FOXP3 (1-177) (encompassing only the N-terminus of FOXP3) (Figure 1A). They were coated on a chip for SPR experiments. It was found that P60 peptide bound with high affinity to the native FOXP3 protein and also to proteins containing the intermediate region of FOXP3, but not to the deletion mutant containing only the N terminus part of FOXP3 (Figure 1B). Thus, these results suggest that P60 is interacting with a region located between aminoacids 177 and 331, which includes the zinc finger (ZF domain; a.a. 200–223), the leucine zipper-like motif (LZ domain; a.a. 240–261) as well as the already described AML1-interacting domain of FOXP3 (located between a.a. 278 and 336) [18].

**Figure 2:**
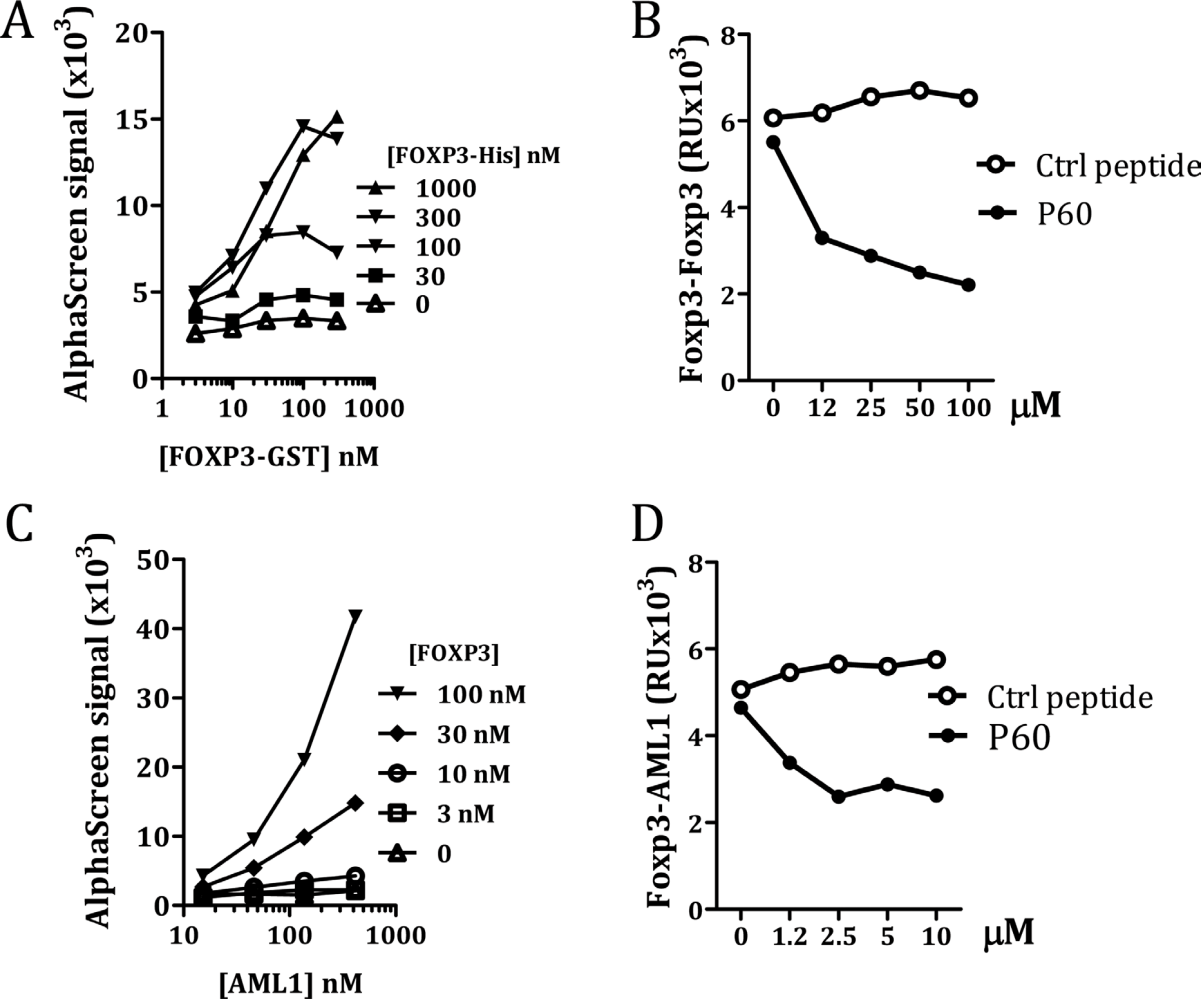
Measurement of FOXP3 homodimerization (**A** and **B**) and FOXP3/AML1 interaction (**C** and **D**) by AlfaScreen. Cross titration experiments to optimize protein concentrations (A and C). Effect of different concentrations of P60 or a control peptide on FOXP3 homodimerization (B) and FOXP3/AML1 interaction (D).

AML1 is required for IL-2 and IFN-γ gene expression in conventional CD4+ T cells and its interaction with FOXP3 is needed for the immunosuppressive activity of Tregs [18]. On the other hand, it has been described that the leucine zipper region is necessary and sufficient to mediate FOXP3 homo-dimerization, which is required for its function as a transcriptional regulator [13, 15, 20]. Thus, since P60 binds to this FOXP3 intermediate region, we analyzed if it could act as a decoy molecule to inhibit FOXP3 homodimerization or the FOXP3-AML1 heterodimerization. The capacity of FOXP3 to homodimerize was measured using the oxygen tunneling assay platform AlphaScreen^™^ (Perkin Elmer). Previous protein-cross titration experiment using different concentrations of FOXP3-6His (from 1000 to 0 nM) and GST-FOXP3 (from 300 to 0 nM) allowed us to define the optimal protein concentrations to analyze FOXP3 homodimerization (Figure 2A). Using this assay (GST-FOXP3 at 40nM and FOXP3-His at 300 nM) we found that peptide P60, but not the control peptide, was able to impair FOXP3 dimerization in a dose dependent manner (Figure 2B). We also tested if GST tagged FOXP3 was able to interact with AML1 protein tagged with 6 His at the N-terminus. As shown in the cross titration experiments in Figure 2C, a specific FOXP3-AML1 heterodimerization was visualized as an increase in the AlphaScreen signal. Interestingly, P60 was able to reduce the signal in a dose dependent manner (Figure 2D), suggesting that binding of P60 to the intermediate region of FOXP3 alters its capacity to interact with AML1.

### Identification of P60 point variants with higher affinity to bind FOXP3 and inhibit FOXP3 homodimerization and FOXP3-AML1 heterodimerization

**Figure 3:**
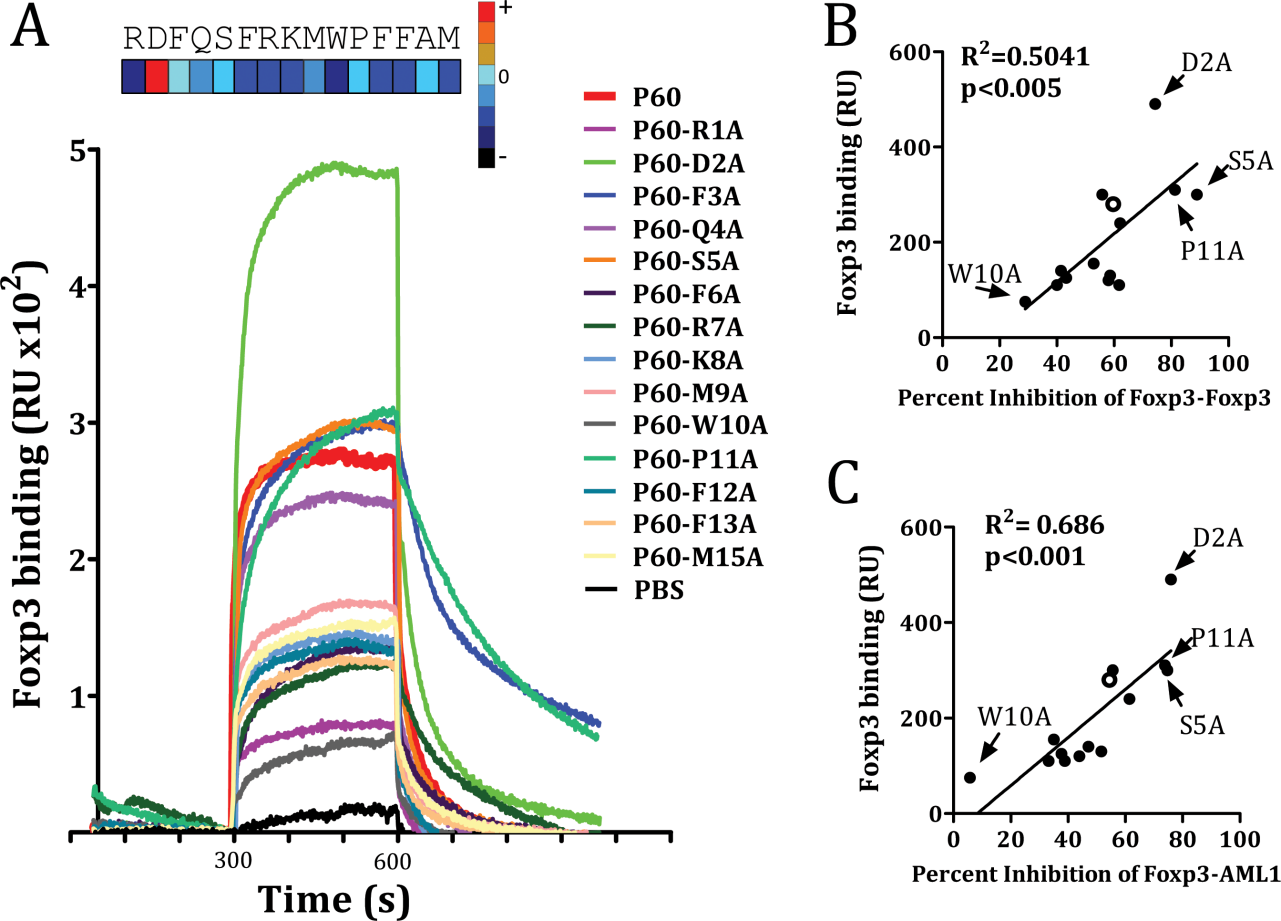
FOXP3 binding of native P60 and each possible point mutation to alanine is measured by SPR (**A**) FOXP3 was coated into the chip and peptides were injected at different concentrations 10 mM. Sensograms obtained for each particular peptide are plotted. Correlation between the capacity to bind FOXP3 and the ability to inhibit FOXP3 homodimerization (**B**) or FOXP3/AML1 interaction (**C**). Data obtained for native P60 is plotted as an empty circle. Relevant point mutations are also identified.

Previous experiments using N-terminus or C-terminus truncated versions of P60 indicated that the 15-mer peptide was required for its activity to inhibit Treg cells [19]. Deletion of 1 aminoacid at the C-terminus or the N-terminus of P60 significantly reduced *in vitro* Treg inhibition (data not shown). Thus, we conducted an alanine scanning program with the 15-mer peptide P60 to identify those residues which contribute to the stability of P60-FOXP3 interactions. Hence, 14 peptides were synthesized by substitution of each individual P60 residue by Ala and subsequently tested for their capacity to bind FOXP3 in SPR assays. It was found that substitution at positions 1, 6, 7, 8, 10, 12, 13 and 15 (residues R, F, R, K, M, W, F, F and M respectively) had a detrimental effect on the binding to FOXP3. Substitution at positions 3, 5 and 11 (residues F, S and P respectively) were permissive or slightly improved the binding. But surprisingly, substitution of aspartic at position 2 by alanine significantly improved the binding capacity to FOXP3 (Figure 3A).

We then analyzed the effect of each mutation on the capacity of P60 to inhibit homodimerization as well as heterodimerization with AML1. A significant correlation was found between their FOXP3-binding capacity and the ability to inhibit FOXP3 homodimerization (Figure 3B), as well as FOXP3-AML1 heterodimerization (Figure 3C). Indeed, peptide mutants such as P60-D2A, P60-S5A and P60-P11A inhibited both FOXP3 homodimerization and FOXP3-AML1 interaction, whereas mutants such as P60-W10A, with a poor FOXP3 binding capacity, did not.

### Identification of P60 point variants with higher capacity to inhibit Treg activity

**Figure 4:**
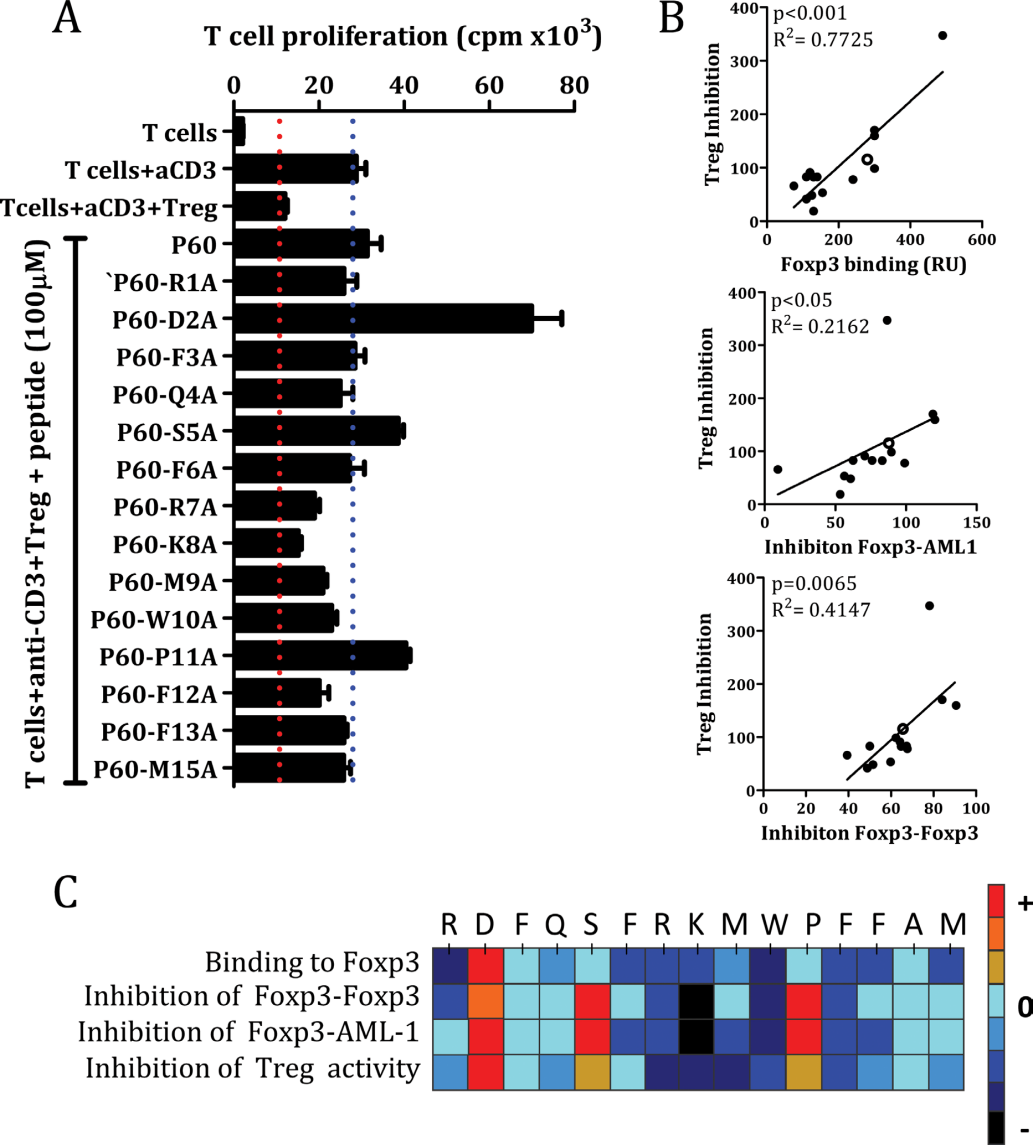
Inhibition of Treg activity by the original P60 and the mutated peptides (**A**). Correlation between Treg inhibitory activity and the capacity to bind FOXP3, disrupt FOXP3 homodimerization or FOXP3-AML1 heterodimerization (**B**). Hot spot summarizing the effect of each point mutation by alanine in the indicated assays (**C**).

Results shown in Figures 1–3 correspond to cell-free assays where peptides can interact directly with its corresponding protein partner. However, FOXP3 binding peptides need to enter into the cell in order to inhibit FOXP3 functions [19]. Once we identified some mutants with higher capacity to bind FOXP3, we studied their capacity to inhibit the suppressor activity of natural Tregs *in vitro*. Thus, we activated effector T cells with anti-CD3 monoclonal antibody in the presence of Tregs, and analyzed the capacity of each peptide to restore T cell proliferation inhibited by Treg cells. It was found that alanine mutations at position 2, 5 and 11 overcame effector T cell proliferation inhibited by Treg, whereas mutations at positions 7, 8, 9, 10 or 12, presented less inhibitory capacity than P60 (Figure 4A). Interestingly, we found a significant correlation between peptide ability to inhibit Treg activity and their capacity to bind FOXP3 (in SPR assays) (*p* < 0.001), or inhibit FOXP3 homodimerization (*p* = 0.0065) or FOXP3-AML1 heterodimerization (*p* < 0.05) (Figure 4B). Indeed, when results from the point-variant screening are represented as a heat map, it is clear that introduction of an alanine at positions 2, 5 and 11 improved activity of the original P60, whereas alanine mutations at positions 1, 7, 8, 9, 10 and 12 are detrimental (Figure 4C).

**Figure 5:**
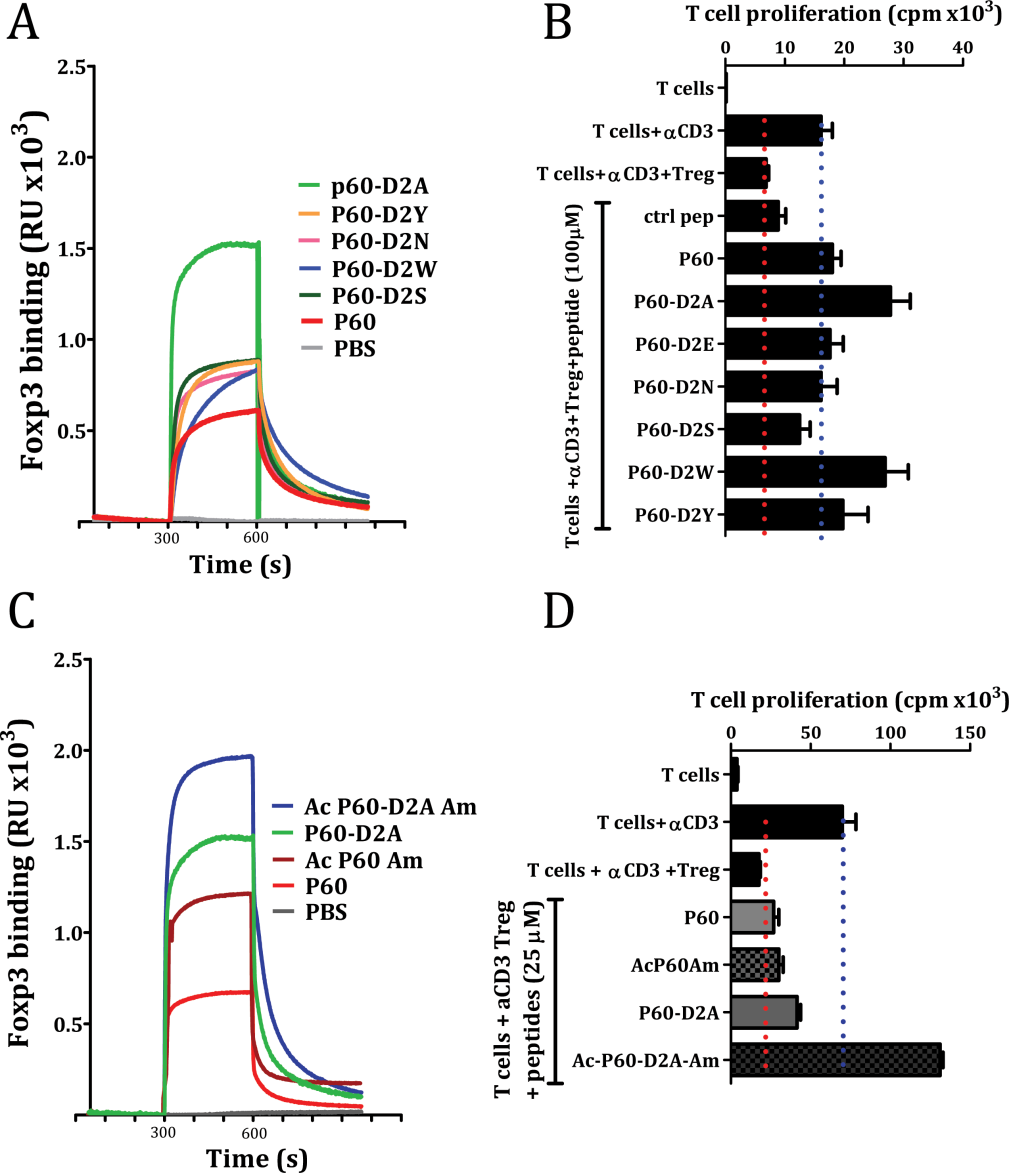
Effect of point mutation at position 2 of the native P60 (**A** and **B**) or the acetylation and amidation of the indicated peptides (**C** and **D**) on their capacity to bind FOXP3 (A and C) or on the inhibition of Treg cells in a cell based bioassay (B and D).

Due to the significant improvement of Treg inhibitory activity in P60-D2A mutant, we analyzed the effect of other aminoacids at this position. Tyrosine (Y) and serine (S) were selected because of their effectiveness in producing high affinity interactions when substituted into the complementary-determining regions (CDRs) of synthetic antibodies [21], and asparagine (N) and tryptophan (W) were selected to span the hydropathy range [22]. SPR analysis showed that all these mutations slightly improved the initial P60-binding capacity to FOXP3, without reaching levels exhibited by P60-D2A (Figure 5A). This activity correlated with their capacity to inhibit Treg effects, except for P60-W2A, which despite its lower binding capacity that P60-D2A, it induced a similar increase in T cell proliferation (Figure 5B).

Originally, P60 was identified by biopanning using a phage-displayed random 15-mer peptide library, where the peptide sequence is expressed within the phage surface protein pIII and it is flanked by other aminoacids. Thus, to mimic this situation, we tested the effect of N-terminal acetylation and C-terminal amidation on peptides P60 and P60-D2A. In both cases, these modifications improved their FOXP3-binding capacity (Figure 5C), and, in the case of P60-D2A, even improved its capacity to inhibit Treg activity. Indeed, when we tested the effect of the peptides at 25μM, which is suboptimal for P60 activity, the acetylated/amidated peptide Ac-P60-D2A-Am showed a strong capacity to improve proliferation of effector T cells in response to anti-CD3 stimulation in the presence of Treg (Figure 5D).

### Effect of double and triple mutations on the inhibitory capacity of the original P60 peptide

**Figure 6:**
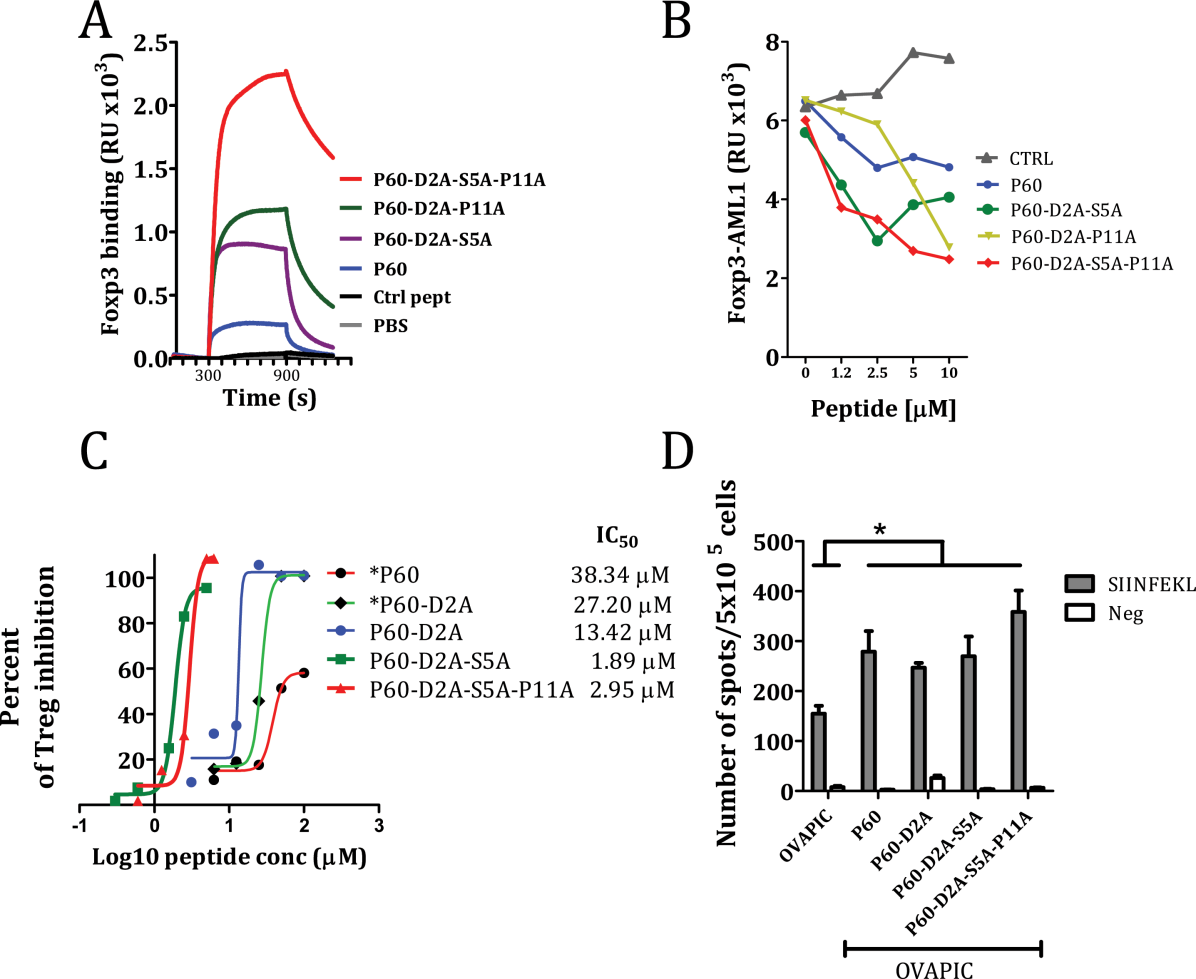
Effect of selected double and triple mutations on the native P60 on the capacity to bind FOXP3 (**A**), inhibit FOXP3/AML1 interaction (**B**), to inhibit Treg cell activity in an *in vitro* cell based bioassay (**C**) or to improve *in vivo* the immunogenicity of ovalbumin (**D**). The number of IFN-gamma producing cells specific for the SIINFEKL peptide in mice immunized with ovalbumin + poly I:C and *in vivo* treated with the indicated peptides was analyzed by ELISPOT. Asterisk are included when peptides are not acetylated and amidated at their ends (*P60 and *P60-D2A).

After analyzing the effect of mutations at positions 2, 5 and 11, we tested if combined mutations would result in a sum of their relative better efficiencies for FOXP3 binding and Treg inhibition. N-terminal acetylation and C-terminal amidation was included in the new mutant peptides P60-D2A-S5A, P60-D2A-P11A and P60-D2A-S5A-P11A and tested them in SPR (at 2.5 μM) for their capacity to bind to FOXP3. We found that the peptide containing double mutations at position 2 and 5 as well as at 2 and 11 improved FOXP3 binding of the peptides. Moreover, the triple alanine mutation improved very significantly the FOXP3 binding capacity. (Figure 6A). When we tested the activity of these peptides to inhibit FOXP3-AML1 dimerization we found that peptide P60-D2A-S5A-P11A as well as P60-D2A-S5A, significantly improved the capacity of the original P60 peptide to inhibit this interaction (Figure 6B). But more interestingly, these peptides containing double and triple mutations significantly improved the capacity of P60 to inhibit Treg activity (from an IC_50_ of 38.34 μM for the original P60 to 1.89μM and 2.95 μM for P60-D2A-S5A and P60-D2A-S5A-P11A respectively, Figure 6C).

Downregulation of Treg suppressor activity *in vivo* may be beneficial to enhance the immunogenicity of a vaccine [19]. We tested if immunization with OVA protein plus poly I:C, which induces low numbers of CD8^+^ T cells specific for the inmunodominant CD8 T cell epitope SIINFEKL, might be improved by the *in vivo* administration of peptide P60 or their mutants. C57BL/6 mice immunized i.v. at day 0 with 1 nmol of OVA plus 50 μg poly I:C and treated with the peptides at days 1, 2 and 3 (100 μg/day i.p. in saline) had a significantly higher number of IFN-γ producing cells in response to ovalbumin or the SIINFEKL peptide than mice treated with saline. However, no clear differences were seen between P60 and the optimized peptides (Figure 6D), suggesting that the *in vivo* pharmacokinetics of the peptides may limit the improved Treg inhibitory capacity observed in the *in vitro* experiments.

### Molecular modelling and pharmacophore generation

**Figure 7:**
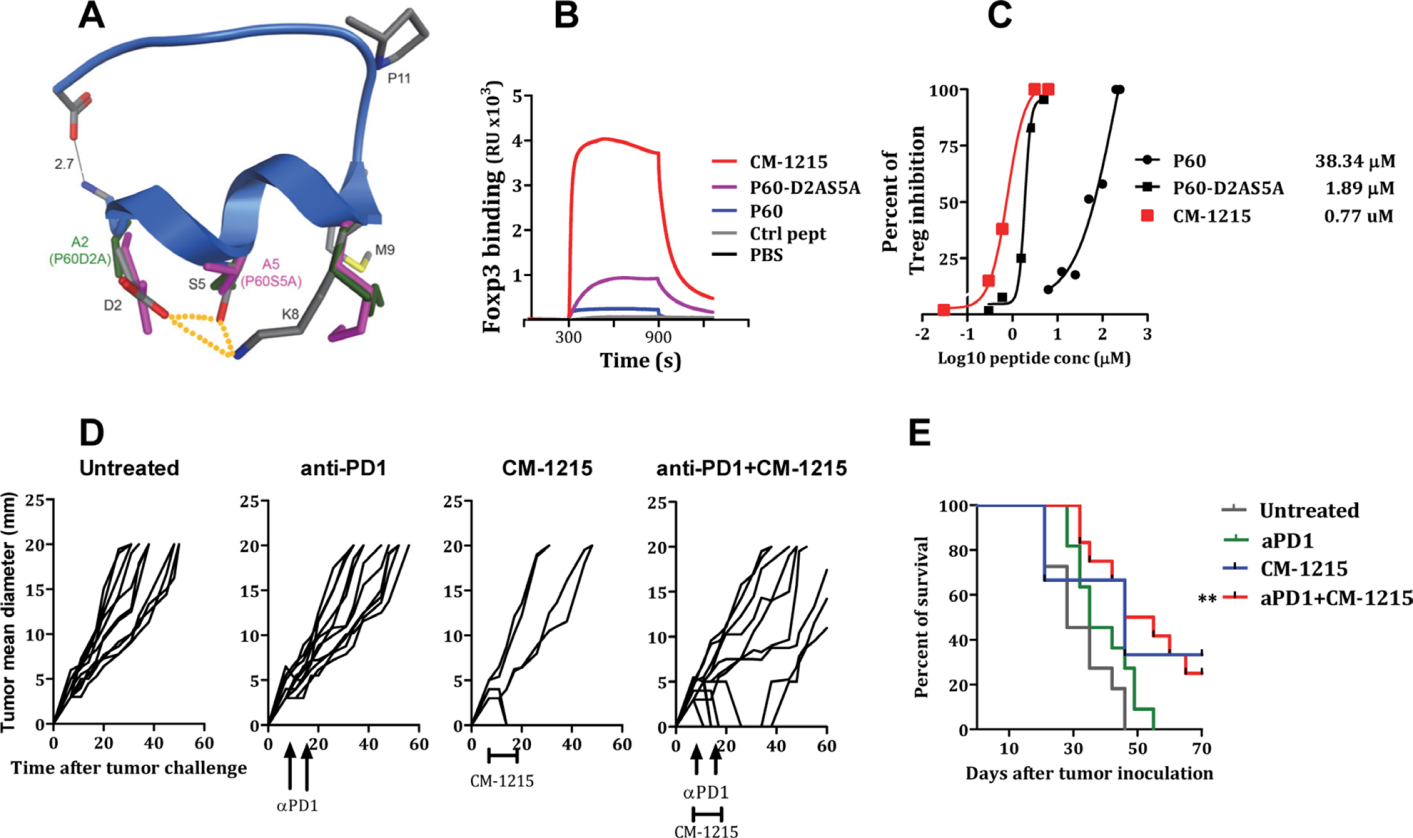
Cyclization of peptide P60-D2AS5A improved FOXP3 binding capacity and Treg inhibitory activity (**A**) Ribbon structure of P60 with alpha-helix from R1 to M9. Element-coloured residues of P60 with the pairwise interactions between D2 and S5, S5 and K8 and D2 and K8 (dotted orange lines). P60-D2A (green) and P60-S5A (pink) side chains of D2 (A2), S5 (A5) and K8. Each alanine mutation releases the side chain of K8, which orients towards M9. (**B**) Surface Plasmon resonance sensograms analysing interaction of the indicated peptide with FOXP3 coated into the chip. (**C**) Percentage of inhibition of Treg activity by the indicated peptides. (**D**, **E**) Treatment efficacy of anti–PD-1 antibody in combination with CM-1215. Hepa-129 cells (10^6^ cells/mouse), were injected subcutaneously (sc) in C3H/HeN mice (*n* = 6). Ten days later, when the tumor reached 5 mm in diameter, mice were treated i.p. with anti-PD1 antibodies (at days 7 and 14) alone, in combination with CM1215 (1 daily administration from day 10 to day 20) or left untreated. (D) Each curve represents tumor mean diameter for an individual mouse. (E) Kaplan–Meier plots of mouse survival. The group of mice treated with anti–PD+ CM-1215 was compared with the rest of the groups with the log-rank test. **P < 0.01.

In order to understand the beneficial impact in FOXP3 binding of the D2A mutation (and to a lesser extent the S5A mutation), a 3D model for P60 and the mutated peptides was generated. Analysis of the P60 structure reveals that the side chain of K8 interacts with and holds up the side chain of D2 and S5 through a hydrogen bond/ionic network (Figure 7A). The presence of the positively charged amino acid K8 is critical for P60 binding to FOXP3 and for the Treg inhibitory activity of P60 (Figure 4). Indeed, whereas substitution of K8 by histidine (P60-K8H) or by arginine (P60-K8R) were well accepted or even improved the binding to FOXP3, a mutation of K8 by E (peptide P60-K8E) dramatically inhibited the binding to FOXP3 (Supplementary Figure 1). The negatively charged carboxylate group of D2 does not seem to be involved in a key direct interaction with any FOXP3 residue counterpart, however, it might affect the capacity of K8 to interact with FOXP3. Thus, in contrast to what is observed in the original P60, the observed hydrogen bond network is not conserved in the mutated P60-D2A, P60-S5A, P60-S5K and P60-S5Y peptides, and the side chain of K8 orients towards M9 (Figure 7A). In fact, molecular dynamic simulations of these peptides shows a hydrogen bond occupancy of 95.5% (in P60) and 0% (in P60-S5A, P60-S5K and P60-S5Y) for the interaction between the side chains of D2 and K8 and a hydrogen bond occupancy of 7.6% (P60) and 0.03% (P60-D2A) for the corresponding side chains of S5 and K8 (not shown). This analysis suggests that the orientation of K8 towards the side chains of D2 and S5, while being enabling for FOXP3 binding, is not as optimal as the orientation adopted by the K8 side chain when released from this hydrogen bond/ionic pairing.

### Effect of peptide cyclization on peptide stability and its activity to inhibit Tregs

Peptide-based drugs, despite their many advantages, are often limited by their poor bioavailability and susceptibility to degradation within the body. Peptide cyclization has been used as a strategy in the pharmaceutical industry for stabilizing and locking the conformation of small peptides. The 3D structure prediction of peptide P60 reveals that the N and C termini are in close proximity (predicted distance between nitrogen at NH terminal and oxygen in carboxy terminal is 2.7 Å, Figure 7A). This suggests that P60 or its derivatives might be amenable to backbone cyclization without a change in the structure of the peptide. Backbone cyclization could enhance the stability of the peptide and resistance to enzymatic degradation and hence improve the pharmacodynamic properties of the molecule. Therefore, we synthesized the backbone cyclic P60-D2AS5A mutant (named CM-1215) with the aim of producing a stabilized drug lead. The metabolic stability of the linear peptide P60-D2AS5A and the cyclic version of the peptide CM-1215 was evaluated in human and mouse liver microsomes after 20 min of incubation at a concentration of 1 μM. The linear peptide showed very poor microsomal stability in human and mouse species (0% remaining); thus, estimated half-life, according to a plausible first order kinetics, is < 2.5 min. However, macrocyclization of the peptide improved microsomal stability (6.2% and 12% remaining, in human and mouse respectively), with an estimated half-life of around 8 min (Table 1). Interestingly, cyclization of the peptide improved very significantly its capacity to bind to FOXP3 (Figure 7B, measured by SPR), and also reduced the IC50 to inhibit Treg activity *in vitro* to 0.77 μM (Figure 7C). In order to evaluate *in vivo* the potential activity of CM-1215 as a Treg inhibitor and as an antitumor agent, we administered the peptide to mice bearing s.c. implanted tumors. Thus, Hepa-129 cells were injected s.c. in C3H/HeN mice. We chose this model because flow cytometry analyses of tumor infiltrates demonstrated a high proportion of Treg. Indeed up to 22% of tumor infiltrating CD4 T lymphocytes express FOXP3, suggesting an important contribution of these cells in tumor escape (Supplementary Figure 2A). Moreover, it was found that a significant proportion of Hepa129 tumor cells express PDL1 *in vivo* (Supplementary Figure 2B). Similarly, a significant number of tumor infiltrating CD4+ve and CD8+ve lymphocytes express PD-1, suggesting that inhibition of PD-1 might be a good strategy in this tumor (Supplementary Figure 2C and 2D respectively).

**Table 1:** Metabolic stability of the peptides

	Human Liver Microsomes			Mouse Liver Microsomes		
Peptide	T1/2 (min)	% Remaining at 20 min	% Remaining at 60 min	T1/2 (min)	% Remaining at 20 min	% Remaining at 60 min
P60	N.D.	N.D.	N.D.	N.D.	N.D.	N.D.
P60-2A	< 2.5	0	0	< 2.5	0	0
CM-1215	5.30	6.20	0	8	12	0
CM-1315	61.7	48.7	46.1	63.8	62.7	47.7

Seven days after Hepa-129 cells injection, when the tumor reached 5 mm in diameter, mice were treated with CM-1215 (from days 7 to 16, 50μg/day/mouse) or anti-PD1 antibodies (50 μg/mice) at days 7 and 14. A group of mice treated with anti-PD1 received also 10 consecutive i.p. administrations of CM-1215 from days 7 to 16 (50 μg/day/mouse). Anti-PD1 administration at these doses did not show a significant antitumor effect. CM-1215 administration alone, although it has some beneficial effect on the percentage of survival, this response did not reach statistical significance with respect to untreated control mice (*p* = 0.061). But importantly, when combined with anti-PD1, CM-1215 exhibited antitumor activity (*p* < 0.001) (Figure 7D–7E).

### Effect of substitution of L-aminoacids by D-aminoacids on P60 derived peptides on its activity to inhibit Tregs

Unfortunately, the clinical potential of P60 derived peptides, and specially the optimized cyclic peptide CM-1215 may be diminished by its susceptibility to proteolytic degradation. It is therefore of interest to develop a biologically active version of CM-1215 more resistant to proteases. The substitution of one or more L-amino acids with D-amino acids might yield peptides that are resistant to proteolytic degradation but retain biological activities [23–25]. We first synthesized the linear versions of peptides containing D-aminoacids at positions 1, 2, and 15. N-terminal acetylation and C-terminal amidation was also included in these peptides. The peptides were tested on their capacity to bind FOXP3, abrogate FOXP3/AML1 interaction and inhibit Treg activity. We found that introduction of a D-Alanine at position 2, did not affect the FOXP3 binding capacity, nor the inhibition of FOXP3 dimerization or FOXP3/AML1 interaction (Supplementary Figure 3A–3C respectively). However a slight increase in T cell proliferation was observed when P60-D2dA was tested on the Treg suppressor assay (Supplementary Figure 3D). Thus, we introduced this D-alanine at position 2 in a new version of the cyclic peptide, named CM-1315. Interestingly, CM-1315 showed a significantly higher metabolic stability in human and mouse liver microsomes (48.7% and 62.7% remaining at 20 minutes, in human and mouse liver microsomes respectively), with an estimated half-life of around 60 minutes (Table 1.)

### *In vivo* administration of CM-1315 improves immunogenicity of AH1 peptide vaccination leading to protection against CT26 tumor challenge

**Figure 8:**
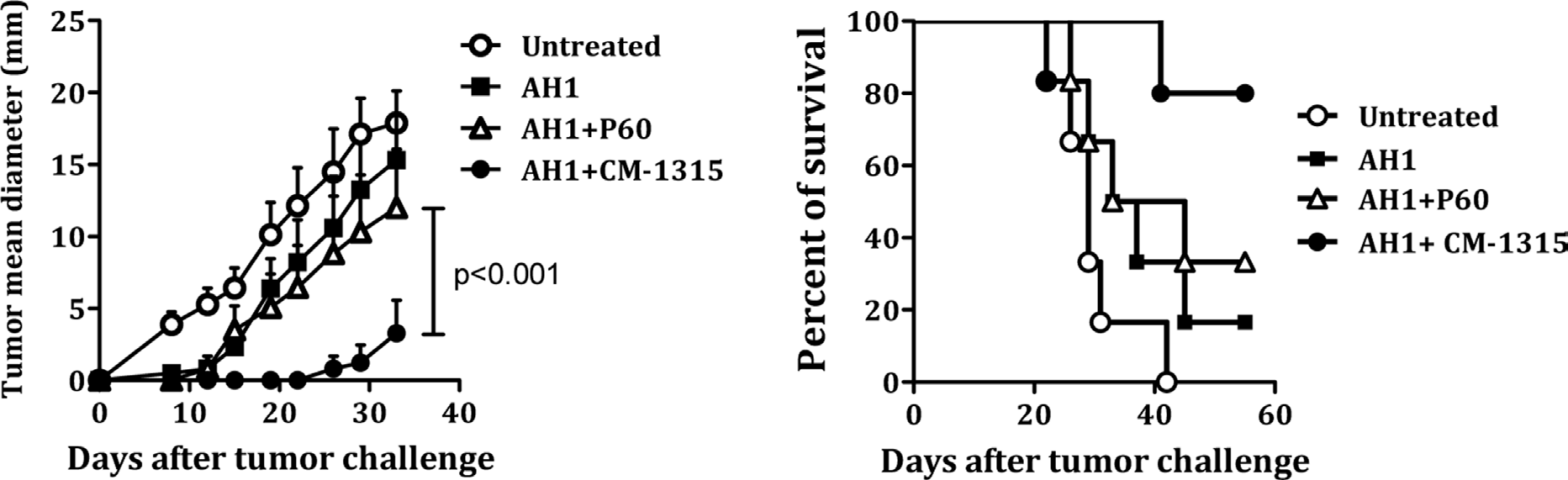
*In vivo* administration of CM-1315 improves vaccine efficacy BALB/c mice were immunized with AH1 peptide emulsified in IFA and treated with saline or with P60 or CM-1315 during 10 days (10 nanomol/mice/day). Ten days later, mice were challenged s.c. with 10^6^ CT26 tumor cells. Mean tumor volume for each group (**A**) and survival curves (**B**) are plotted.

Immunization of BALB/c mice with peptide AH1 emulsified in IFA is unable to induce a protective CTL response against challenge with CT26 tumor cells [19, 26]. However, depletion of CD25+ Treg cells with anti-CD25 antibodies before immunization with peptide AH1 resulted in the induction of a long lasting anti-tumoral immune response [27]. In a previous work we found that *in vivo* Treg inhibition with P60 in combination with vaccination with AH1 protected mice from CT26 tumor challenge. In those experiments, BALB/c mice were immunized with peptide AH1 emulsified in IFA and treated daily with saline or with 50 nanomol/mice/day of peptide P60 i.p. from day 0 to day 9 after immunization. To study the efficacy of CM-1315, our optimized version of P60, mice immunized with AH1 were treated with 10 nanomol/mice/day of P60 or CM-1315 and challenged at day 10 by s.c. injection with 10^6^ CT26 tumor cells (instead of 5 × 10^5^ cells used in our previous study). As control, groups of mice were injected with AH1 alone or left untreated. We found that only animals treated with CM-1315 remained protected against challenge with CT26 tumor cells (Figure 8), suggesting that P60 optimization, also enhanced the *in vivo* Treg inhibitory activity, improving the immunogenicity of the AH1 peptide vaccine.

### CM-1315 improves the effector functions of conventional T cells

Results obtained from Figures 4 and 5, where the addition of the peptides P60-D2A, P60-S5A and P60-P11A not only prevented the inhibition of Treg but also boosted the basal Tconv proliferation detected in the absence of Treg, are remarkable. FOXP3 is critical for the development and function of Tregs, but also, FOXP3 can be transiently expressed in effector T cells after TCR stimulation leading to hyporesponsiveness of the effector T cell. In a previous work [28] we found that inhibition of NFAT-FOXP3 interaction by a synthetic peptide augmented T cell proliferation and the production of cytokines by conventional effector T cells, suggesting that this transient expression of FOXP3 may reduce the effector and proliferative capacity of T cells. We speculate that the Foxp3 inhibitory peptides may work at two levels: (i) reducing Treg function and (ii) releasing the brake of FOXP3 transient expression on effector T cells. Thus, we studied the effect of CM-1315 in T cell proliferation and IL-2 and IFN-γ production in effector T cells in response to anti-CD3 stimulation. The original P60, the optimized peptide CM-1315 and a control peptide were tested. We found that FOXP3 peptide inhibitors augmented T cell proliferation as well as cytokine production, being CM-1315 more active than P60, indicating that FOXP3 inhibitory peptides are directly affecting the activity of conventional T cells (Supplementary Figure 4). We also studied the effect of the peptides directly in Tregs. Thus, freshly isolated Treg cells were pre-treated with the peptides for 24 h, and after extensive washing, they were used in conventional Treg suppression assay in coculture with effector T cells and anti-CD3 stimulation. We found that incubation of Treg with CM-1315 at 20 μM for 24 hours was able to inhibit significantly the suppressive activity of Treg. At this concentration, the original P60 was not able to inhibit significantly Treg cells, although some reversal of suppression was observed (Supplementary Figure 5).

We also studied the effect of CM-1315 peptide *in vivo* to improve the immune response after immunization with AH1 peptide in naïve mice and in mice depleted of CD25+ T cells. In this experiment, depletion of CD25+ T cells improved the number of AH1-specific IFN-γ producing cells. But more interestingly, the *in vivo* administration of CM-1315 to mice depleted of CD25+ T cells significantly improved this response, suggesting that the peptide is also directly affecting the functionality of effector T cells in response to TCR stimulation (Supplementary Figure 6).

## DISCUSSION

It is widely acknowledged that inhibiting Treg-cell function in patients with cancer is essential to improve the efficacy of anti-tumoral therapies. Several strategies have been proposed to deplete or neutralize Treg activity [29–31]. However, these strategies lack of high specificity and might eliminate both effector T-cells and Treg cells. Targeting FOXP3, a transcription factor essential for the specification and maintenance of Treg cells, is an alternative to control Treg activity. The capacity of FOXP3 to bind DNA is critical for its functionality; however, it is clear that FOXP3-DNA interactions are assisted by FOXP3 cofactors and by multimerization. [32]. FOXP3 orchestrates a transcriptional network in collaboration with a large number of cofactors, and thus, regulation of the members of the FOXP3 interactome may offer opportunities for the development of new treatments for autoimmune diseases, cancer or infectious diseases. In a previous work, using a phage displayed peptide library, we identified a 15-mer synthetic peptide, P60, able to enter into the cells, bind to FOXP3, and impair Treg activity *in vitro* and *in vivo* [19]. In the present work, we have found that P60 interacts with a region between aminoacids 177 and 331 from FOXP3, which includes the Zinc finger (ZF) and the Leuzin Zipper (LZ) domains as well as the sequence between the LZ and the FKH domain of FOXP3. Interestingly, this region, and in particular de LZ domain, has been described as necessary and sufficient for dimerization of FOXP3 and required for the inhibitory function of Tregs [13, 15, 17, 20]. Also, the region between amino acids 278–336, just immediately after the LZ domain, has been described to interact with the transcription factor AML1, which is needed for thymic T-cell development [33–35] and for IL-2 and IFN-γ gene expression in conventional CD4+ T cells. AML1 interaction with FOXP3 suppresses IL-2 and IFN-γ production by T cells, controls anergy of the cell and is required for the suppressive activity of Treg [18, 36]. We have found that P60 can indeed inhibit FOXP3 homodimerization as well as FOXP3/AML1 interaction, suggesting that the Treg inhibitory activity of P60 may be related to its capacity to impair these protein-protein interactions. Previous works revealed that FOXP3 coiled-coil-mediated homodimerization is essential for Treg function *in vitro* and *in vivo* [13, 15, 17, 20]. Identification of IPEX patient with mutations in this intermediate region of FOXP3 [37] might support the relevance of FOXP3 homodimerization on the activity of Treg cells. We cannot determine the relative contribution of the disruption of FOXP3-AML1 interaction or the FOXP3 homodimerisation on the capacity of P60 to inhibit Treg activity. Furthermore, this intermediate region of FOXP3 has been shown to interact also with FOXP1, an interaction that may play a role in FOXP3-mediated repression of IL-2 production [13]. Further experiments, such as the knockdown of endogenous FOXP1 or AML1, are needed to evaluate the individual role or the relative participation of each interaction on Treg function.

In an attempt to get insights on the role of each residue within P60 sequence and to try to optimize the efficacy of P60, we performed an alanine scanning program with the 15-mer peptide P60 to identify side chains that contribute to the stability of P60-FOXP3 interactions. SPR analyses (to measure peptide binding capacity to FOXP3) and *in vitro* assays to measure FOXP3/FOXP3 and FOXP3/AML1 dimerization was used to help understand the structure–function role of the constituent amino acids in P60. Interestingly, we found a significant correlation between the effect of each point mutation in the FOXP3 binding capacity and their ability to disrupt protein/protein interactions (measured by Alphascreen). But also, a significant correlation was found when compared these biochemical assays with a cell-based assay to measure their biological activity, where other important properties, such as the capacity to enter into the cell through cell membranes, may play a critical role. Indeed, a correlation was found between the effect of each particular mutation on FOXP3 binding, or in protein/protein interactions, with the capacity to inhibit Treg activity. These experiments have led to a better understanding of the importance of spatial organization of key residues in binding interfaces. Indeed, we identified positions 2, 5, 8, 10 and 11 as key elements on P60 activity.

Notably, D2A substitution and to a lesser extend S5A and P11A, significantly improved P60 binding capacity to FOXP3. This substitution enhanced its inhibitory activity over FOXP3 homodimerization and FOXP3-AML1 heterodimerization, as well as its capacity to inhibit Treg activity (see the heat map from Figure 4C). These results suggest that the negative charge at position 2 might decrease peptide binding to FOXP3. Indeed, almost any variation tested at position 2, enhanced FOXP3 binding. Substitution of Serine by Alanine at position 5 also enhanced FOXP3 binding affinity. Interestingly, K8A substitution significantly reduced P60 activity, suggesting that this positively charged aminoacid might play an important role. Indeed substitution of K8 by histidine (P60-K8H) or by arginine (P60-K8R) were also well accepted. Moreover, amino acids flanking the K8 charged residue (RKMW) seem to play a role favouring the binding capacity of the peptide with its interacting partner. Indeed, a single substitution of any these K flanking residues by alanine reduced to ability of P60 to inhibit Tregs.

Other residue, such as Tryptophan at position 10 is also required for FOXP3 interaction. Trp is also relevant since it has been described that cell penetrating peptides (CPPs) are characterized by the presence of this aminoacid on the sequence [38–39]. Proline at position 11, an aminoacid characterized by its rigidity, seems also to be limiting P60 interaction with FOXP3, since its mutation by Ala slightly improved FOXP3 binding and significantly enhanced its capacity to inhibit FOXP3 homodimerization and heterodimerization with AML1, as well as its activity inhibiting Treg activity. It would be postulated that flexibility at this point might favor the docking of the peptide to FOXP3.

Analysis of potential 3D model for P60 and its mutants using in silico techniques allows us to hypothesize that the side chain of K8 interacts with and holds up the side chain of D2 and S5 through a hydrogen bond/ionic network. Indeed, in the mutated P60-D2A and P60-S5A peptides, the observed hydrogen bond network is not conserved and the side chain of K8 orients towards M9. This analysis suggests that the orientation of K8 towards the side chains of D2 and S5, while being enabling for FOXP3 binding, is not as optimal as the orientation adopted by the K8 side chain when released from this hydrogen bond/ionic pairing. This hypothesis might also explain the significant improvement of the inhibitory capacity of peptides P60-D2A and P60-D2A-S5A, substituting D2 and S5 by alanine. It is tempting to postulate that this positively charged K8 residue might interact with negatively charged residue E242 (E243 in human FOXP3) and break the E242-K249 (E243-K250 in human FOXP3) intersubunit salt bridge, which has shown to be essential in FOXP3 homodimerization through the LZ domain [17]. Interestingly, residues flanking this key amino acid K249 (EK249EK) are all charged residues, of which K251 has been shown crucial for regulating the stability of the FOXP3 homodimer. Currently, the crystal of the FOXP3 coiled coil (PDB entry 3HSX) remains unpublished, but taken together this data we have a rational basis for the observed general beneficial role when introducing positively charged amino acids at different positions (e.g. P11). Alternatively, this K8 residue might interact with the region FOXP3 329-332 (DHFK) which is required for FOXP3-AML1 interaction [18]. However, further experiments are needed to evaluate these possibilities.

The 3D structure prediction of peptide P60 also revealed that the N and C termini are in close proximity, suggesting that backbone cyclization could stabilize the structure and even improve the affinity to bind FOXP3. Moreover, as mentioned above, P60 was identified by biopanning using a phage-displayed random 15-mer peptide library, where the peptide sequence is flanked by other aminoacids. Accordingly, N-terminal acetylation and C-terminal amidation on P60 derived peptides improved their FOXP3-binding and its capacity to inhibit Treg activity. Therefore, we synthesized the backbone cyclic P602A5A (CM-1215) mutant with the aim of producing a stabilized drug lead. This cyclization improved microsomal stability and the estimated half-life. Interestingly, cyclization of the peptide improved very significantly its capacity to bind to FOXP3, to inhibit Treg *in vitro*, and more importantly, to delay tumor growth in combination with anti-PD1 antibodies in a model of hepatocellular carcinoma. This improvement might be also due to a better capacity of the modified peptides to penetrate through the plasma membrane, however, we did not found significant differences in cell permeability between the original CFSE labelled P60 and the optimized versions (data not shown). Our results suggest that the improvement in stability was good enough to observe its impact on tumor progression. In an attempt to enhance peptide stability we introduced non natural aminoacids (D-aminoacids) at different positions and found that a D-alanine at position 2 improved very significantly the peptide half-life and its *in vivo* activity in a model of preventive vaccination against challenge with tumor cells. Nevertheless, there is still a room for improvement and additional macrocycles are being explored. CM-1315 macrocycle consist on a head to tail cyclization. We are now testing new cyclization options including new spacers and functional residues that might improve the pharmacokinetic profile of this FOXP3 inhibitory molecule.

Peptides have gained increased interest as therapeutics during recent years. More than 60 peptide drugs have reached the market for their therapeutic use in patients, around 150 novel peptides are currently in clinical trials whereas more than 500 therapeutic peptides are in preclinical development [40]. Despite their poor chemical or physical stability, and their short half-life in plasma, peptides represent an excellent starting point for the design of novel therapeutics. Their specificity, safety and tolerability makes them attractive compared with traditional small molecules whereas their production costs and synthesis feasibility constitute an advantage compared with protein-based biopharmaceuticals. Peptides can be designed to target a broad range of molecules, giving them almost limitless possibilities in fields such as oncology, immunology, infectious disease and endocrinology. Peptides such as cyclosporine (a potent immunosuppressant agent acting by its binding to cyclophilin and inhibiting the phosphatase activity of calcineurin) or desmopressin (an antidiuretic acting by binding to V2 receptors), approved in 1983 and 1978 respectively and included on the World Health Organization's List of Essential Medicines, are good examples of the potential of peptides as a therapeutic tool.

Another alternative to synthetic peptides would be the use of recombinant viral vectors. This tool might permit a better long-term alternative to express the peptides *in vivo*. This approach could be used for peptides not including non-natural modifications such as D-aminoacids or peptide cyclizations. We prepared viral vectors expressing a secretable version of P60. A minigene coding for P60 linked to a leader peptide to favour peptide secretion from the infected cell was designed. The adenoassociated viral vector AAV-P60 expressing the P60 peptide under the control of the hepato-specific promoter alpha 1 antitrypsin was produced and used to treat mice bearing Hepa129 tumors. It was found that 5 out of 12 mice treated with this P60 expressing vector were able to reject tumors, as opposed to 0/6 of mice treated with AAV expressing luciferase as control (Supplementary Figure 7). This experiment opens the possibility to use viral vectors expressing the FOXP3 inhibitory peptides as a therapeutic tool to treat tumors.

In summary, we have found that the Treg inhibitory peptide P60 interacts with the intermediate region of FOXP3 inhibiting homodimerization and its association with AML1. The alanine scan program identified key residues implicated in the interaction of P60 with FOXP3 and allowed us to define a pharmacophoric model for the identification of a cyclic peptide with improved capacity to inhibit Treg cells *in vitro* and with anti-tumor activity *in vivo*. These results may be of help on the design of new therapies against cancer.

## MATERIALS AND METHODS

### Peptide synthesis

Peptides were synthesized by the solid phase method of Merrifield using the Fmoc alternative as previously described [41]. The purity of the peptides was analyzed by HPLC. Cyclic peptides were synthesized by Wuxi AppTech (Shanghai, China).

### Biomolecular interaction analysis by surface plasmon resonance and by AlphaScreen technology

Screening of peptide binding to FOXP3 was performed by surface plasmon resonance (SPR) using ProteOn XPR36 (Bio-Rad, Hercules CA, USA) optical biosensor. Recombinant protein FOXP3-6His and the indicated truncated versions of FOXP3 were produced and purified from E-coli and immobilized covalently onto a GLM sensor chip (Bio-Rad) using sulfo-NHS and EDC (Bio-Rad) coupling reagents. After protein immobilization, chip surface was treated with ethanolamine to deactivate the excess of reactive esters. Individual peptide solutions (1–20 μM) were injected by triplicate in running buffer (Phosphate buffered saline, 0.005% (v/v) Tween 20, pH 7.4) at a flow of 30 μl/min. The interspot signal (obtained in the chip surface not immobilized with protein) was used as reference.

FOXP3/AML1 heterodimerization, as well as FOXP3/FOXP3 homodimerization were analyzed by Alphascreen technology according to the manufacturer's instructions (Perkin Elmer, Benelux) as previously described [28].

### T regulatory cell purification

Isolation of murine CD4^+^CD25^+^, and CD4^+^CD25^−^ T-cells was performed from murine spleen cells by using murine regulatory T-cell isolation kit (Miltenyi Biotec, Bergisch Gladbach, Germany) according to manufacturer's instructions. The purity of the resulting T-cell populations was confirmed to be > 95% by flow cytometry.

### *In vitro* assays for murine T regulatory cell function

Inhibition of murine T regulatory cell function was measured in an *in vitro* assay of T-cell stimulation. CD25+ depleted spleen cells (10^5^ cells/well) from BALB/c mice were stimulated *in vitro* with 2, 5 μg/ml of anti-mouse CD3 antibody (Pharmingen) in the presence/absence of purified Treg cells (10^4^ cells/well). T-cell proliferation was measured 3 days later as previously described [42].

### *In vivo* experiments

Naïve C57BL/6 mice (*n* = 6) received an intravenous administration of ovalbumin (OVA) protein (1 nmol/mouse) plus poly I:C (50 μg/mouse). The indicated groups received an intraperitoneal injection with 100 μg of P60 or P60 derived peptides at days 1, 2 and 3 after immunization. Mice were killed at day seven and splenocytes were obtained for immunological analysis. Cells producing IFN-γ were enumerated by ELISPOT assays (BD-Biosciences) as described [43]. For T-cell responses, splenocytes were stimulated with peptide OVA (257–264, 1 μg/ml) (SIINFEKL peptide) and OVA protein (10 μg/ml).

For tumor rejection experiments, Hepa-129 cells (10^6^ cells/mouse), were injected subcutaneously (sc) in C3H/HeN mice (*n* = 6). Ten days later, when the tumor reached 5 mm in diameter, mice were randomly divided into different experimental groups. A group of mice were treated i.p. with anti-PD1 antibodies (50 μg/mouse). Antibody administration was repeated one week after this first administration. A group of mice treated with anti-PD1 also received the cyclic peptide CM-1215 from days 10 to 20 (50 μg/mice/day). Tumor size, presented as the average of two perpendicular diameters (millimetres), was measured at regular intervals. Mice were sacrificed when the mean tumor diameter was greater than 20 mm. For anti-tumor vaccination experiments, animals immunized s.c. with 50 nanomol/mice of peptide AH1 emulsified in incomplete Freund adjuvant (IFA) [27], were treated daily with 10 nanomol/mice (i.p) of the indicated Treg inhibitory peptide or with saline during 10 days. At day 10, mice were injected s.c. with 10^6^ CT26 tumor cells. Tumor size was monitored twice a week with a calliper and it was expressed according to the formula V = (length × width^2^)/2. Mice were sacrificed when tumor size reached a volume greater than 4 cm^3^. Mice were housed in appropriated animal care facilities during the experimental period and handled following the international guidelines required for experimentation with animals. Institutional ethical committee approved the experiments.

### P60 modelling and alanine scanning

The 3D structure of the P60 peptide was predicted using the *de novo* prediction server PEP-FOLD [44–45]. The model with the best sOPEP energy value was minimized in MOE (MOE 2014.09. Chemical Computing Group, Inc. Molecular Operating Environment, Montreal, Quebec, Canada) using Amber12:EHT as force field and the Generalized Born model as implicit solvation energy. Then, key residues of the minimized P60 structure were sequentially mutated to alanine and minimized under the same conditions than for the P60 peptide to generate the 3D conformations. Finally, for each peptide, a molecular dynamic simulation of 15 ns each was performed with MOE after an equilibration stage of 5 ns with gradual increase of the temperature to the simulation temperature of 300 K (Nosé-Poincaré-Andersen equations of motion and time step of 0.002 ps). The hydrogen bond occupancy (fraction of time that the hydrogen bond is formed) was monitored for the following pairwise side-chain interactions: carboxylate group of D2 with the N-terminal amine of K8, carboxylate group of D2 with the hydroxyl group of S5 and hydroxyl group of S5 with the N-terminal amine of K8.

### Metabolic stability

Metabolic stability was measured by Wuxi AppTech (Shanghai, China). Briefly, peptides (1 μM, 5% MeOH in potassium phosphate buffer) were incubated with human (catalogue no. 452161 from BD Gentest) and mouse (catalogue no. M1000, Xenotech) liver microsomes at 37°C for 10 min. Liver microsomes were at a final assay concentration of 0.7 mg protein/mL. The reaction was started by the addition of 90 μL of NADP cofactor solution and stopped by the addition of 300 μL of stop solution (acetonitrile at 4°C, including 100 ng/mL tolbutamide as an internal standard) after 20 min of incubation. The samples were shaken for 5 min and then centrifuged for 20 min at 1500 g. A 100 μL aliquot of the supernatant was transferred to eight new 96-well plates with 300 μL of HPLC water and centrifuged at 1500 g for LC-MS/MS analysis (Shimadzu LC 10-AD−API 4000). An injection volume of 10 μL was added to a Phenomenex Synergi C18 column eluting with formic acid in water or acetonitrile at a flow rate of 800 μL/min. The percent loss of parent compound was calculated from the peak area ratio of the analyte/internal standard. Compounds and positive controls were tested in duplicate.

### Statistical analysis

Normality was assessed with Shapiro-Wilk W test. Statistical analyses were performed using parametric (Student′s t test and one-way ANOVA) and non-parametric (Mann-Whitney U and Kruskal-Wallis) tests. For all tests a *p* value < 0.05 was considered statistically significant. Descriptive data for continuous variables are reported as means ±SEM. GraphPad Prism for Windows was used for statistical analysis.

## SUPPLEMENTARY FIGURES



## Abbreviations

Treg: T Regulatory cells
FOXP3: Forkhead box P3 transcription factor
P60: a peptide inhibitor of Foxp3
AML1: acute myeloid leukaemia 1 trancription factor
LZ: Leuzin zipper domain
ZF: Zinc finger domain
