# Supplementary material for: Blockage of FOXP3 transcription factor dimerization and FOXP3/AML1 interaction inhibits T regulatory cell activity: sequence optimization of a peptide inhibitor
Source: Oncotarget. 2017 May 13;8(42):71709–24. doi: 10.18632/oncotarget.17845 (PMC5641083; doi:10.18632/oncotarget.17845)
Supplement: Supplementary file 1 [file oncotarget-08-71709-s001.pdf]

Blockage of FOXP3 transcription factor dimerization and FOXP3/AML1 interaction inhibits T regulatory cell activity: sequence optimization of a peptide inhibitor

Supplementary Materials

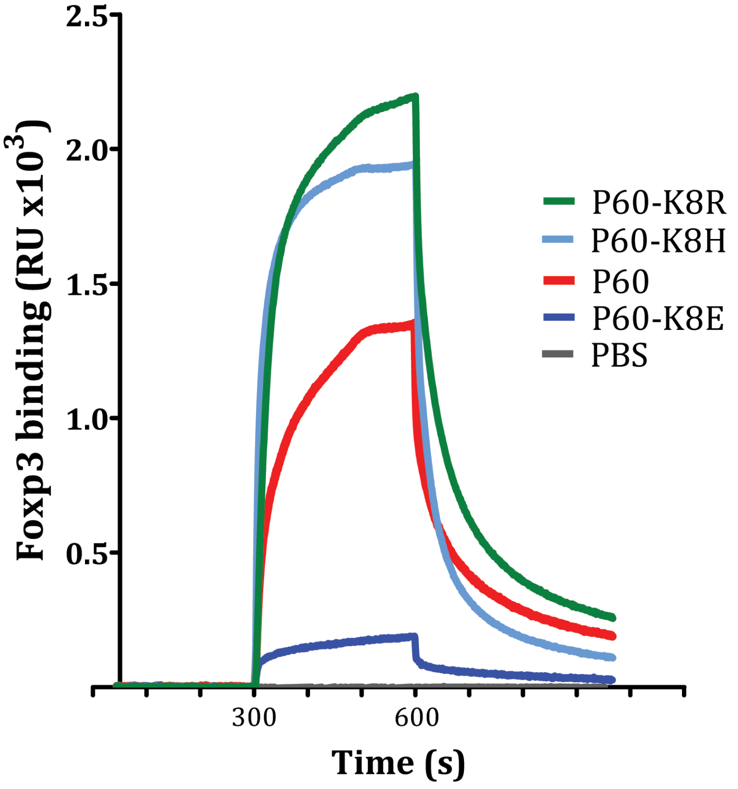

Supplementary Figure 1: Effect of indicated mutations on native P60 at position 8 on the capacity to bind FOXP3 measured by SPR.

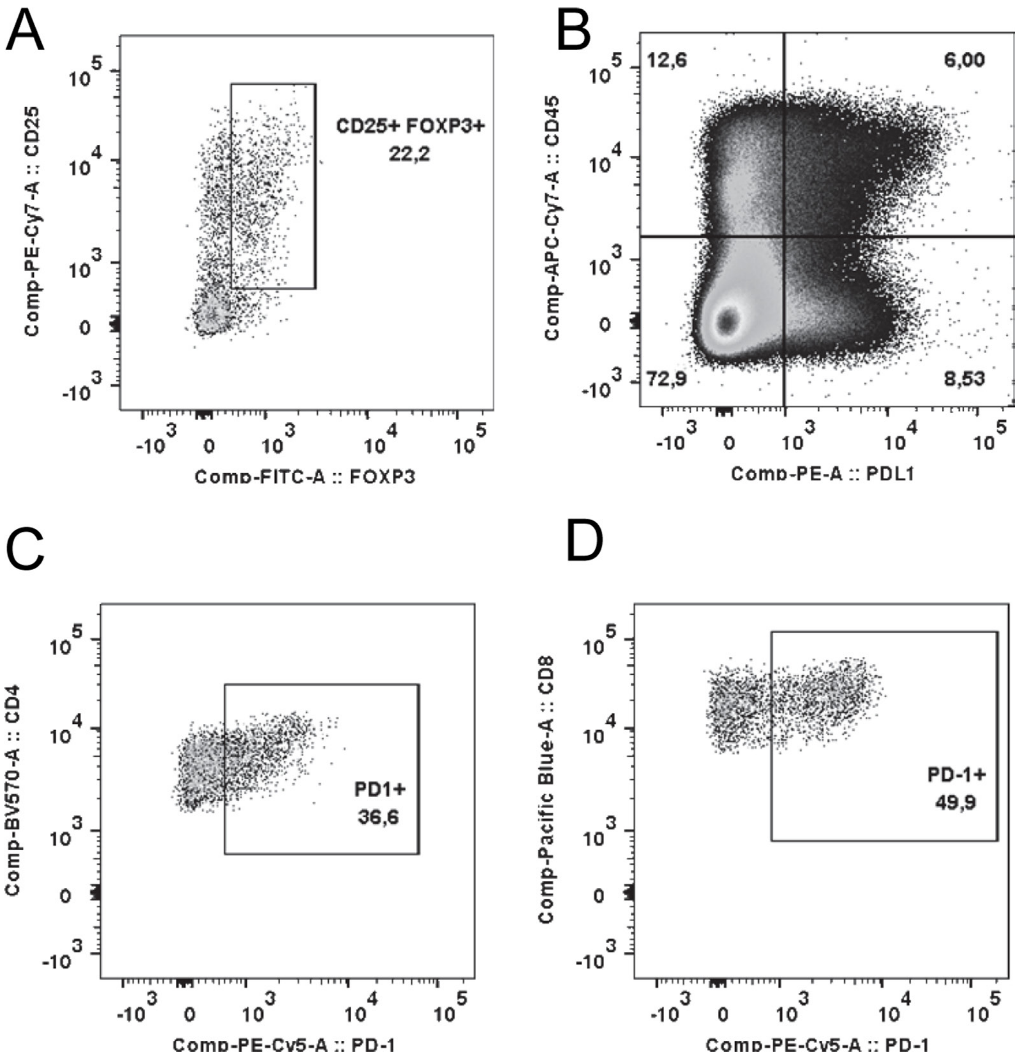

Supplementary Figure 2: Characterization of Hepa129 based tumor model by flow cytometry. Mice were challenged with Hepa129 tumor cells s.c. At day 15, tumors were homogenized and labelled with the indicated antibodies and analysed by Flow cytometry. (**A**) Percentage of CD4+CD25+Foxp3+ cells infiltrating Hepa129 tumors. (**B**) Percentage of CD45-ve PD1+ve and CD45+PD1+ve cells in Hepa129 tumors. (**C** and **D**). Percentage of CD4+vePD1+ve (C) and CD8+ve PD1+ve (D) cells infiltrating Hepa129 tumors.

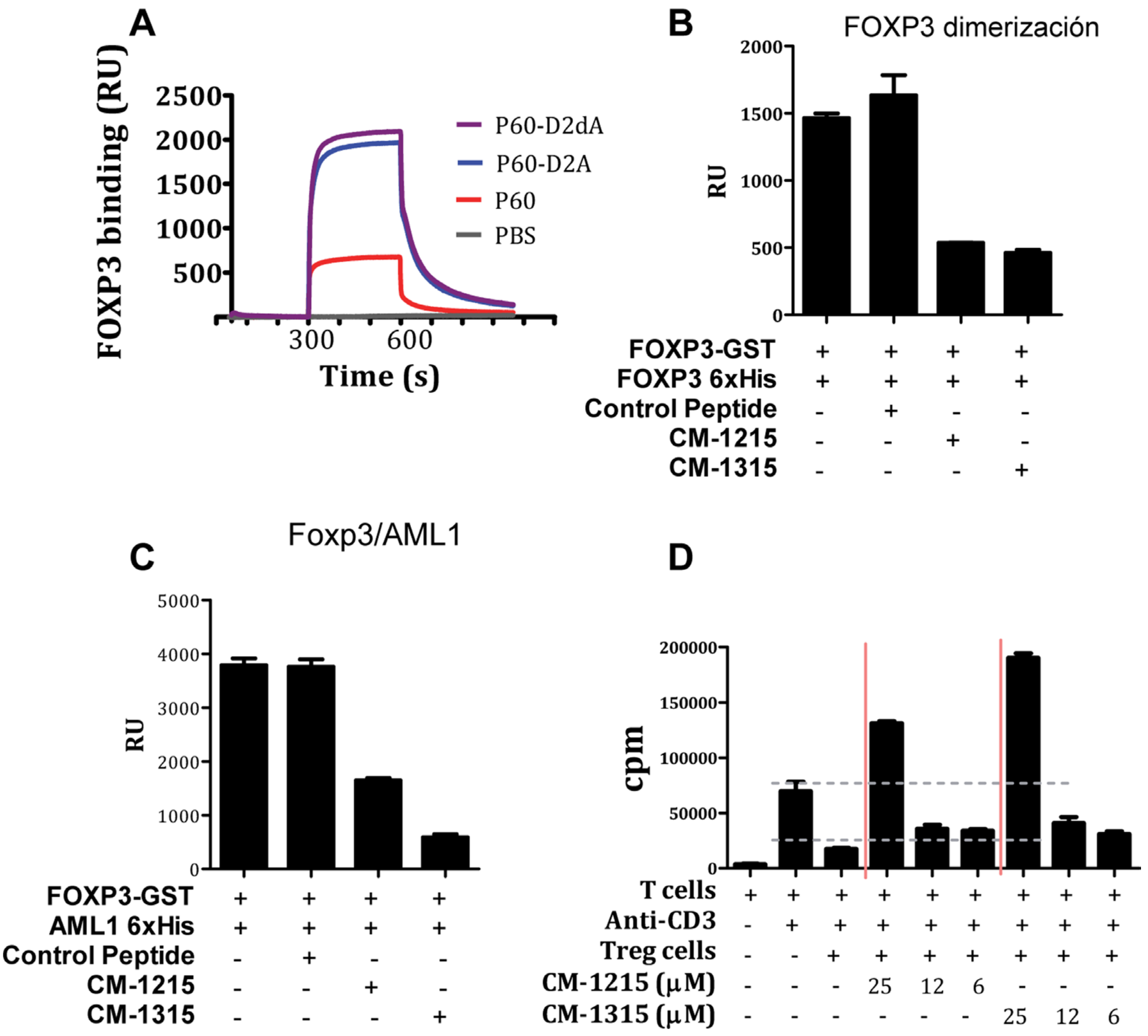

Supplementary Figure 3: Effect of the introduction of D-alanine at position 2 on the capacity to bind FOXP3 (**A**) inhibit FOXP3 dimerization (**B**) FOXP3/AML1 interaction (**C**) or Treg inhibition (**D**).

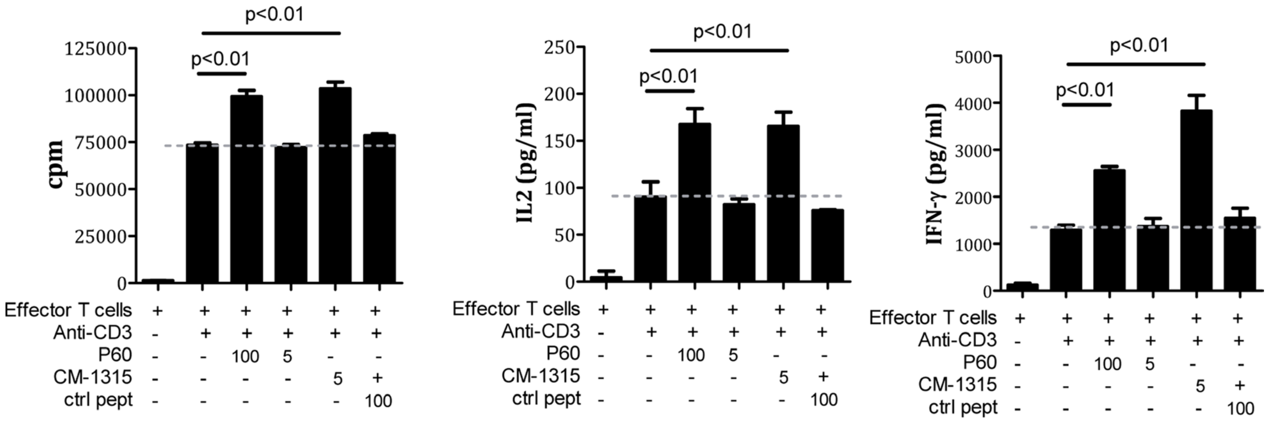

Supplementary Figure 4: Effect of Foxp3 inhibitory peptides in effector T cells alone. CD4+CD25-ve splenocytes were stimulated with anti-CD3 in the presence/absence of the indicated peptide. T cell proliferation (**A**) and IL-2 and IFN-γ (**B** and **C**) production in response to anti-CD3 stimulation was measured by 3H-thymidine incorporation and by ELISA respectively.

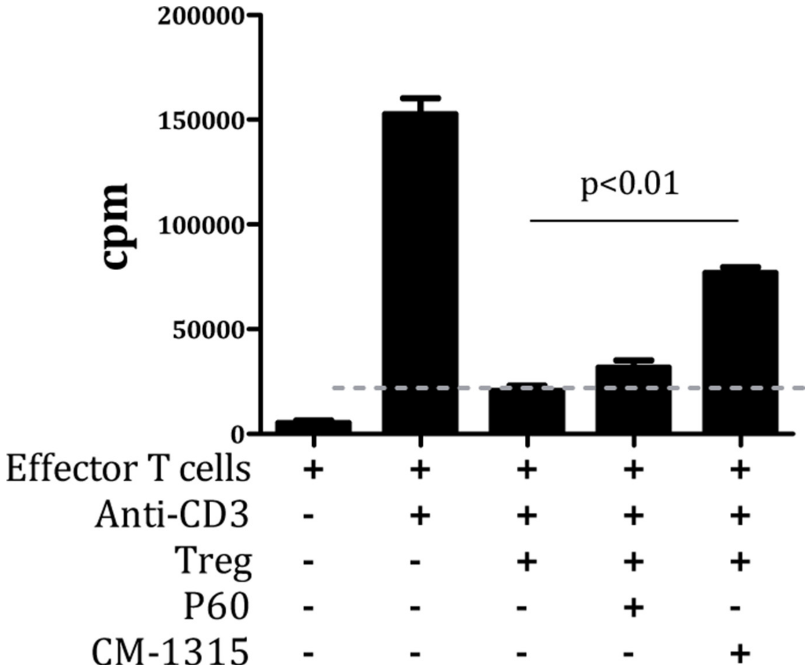

Supplementary Figure 5: Effect of FOXP3 inhibitory peptides in Treg cells. Treg cells were pre-treated with the peptides ant 20 µM for 24 h, and after extensive washing to remove the peptide, they were used in the conventional Treg suppression assay in coculture with effector T cells and anti-CD3 stimulation. T cell proliferation was measured by 3H-thymidine incorporation assay.

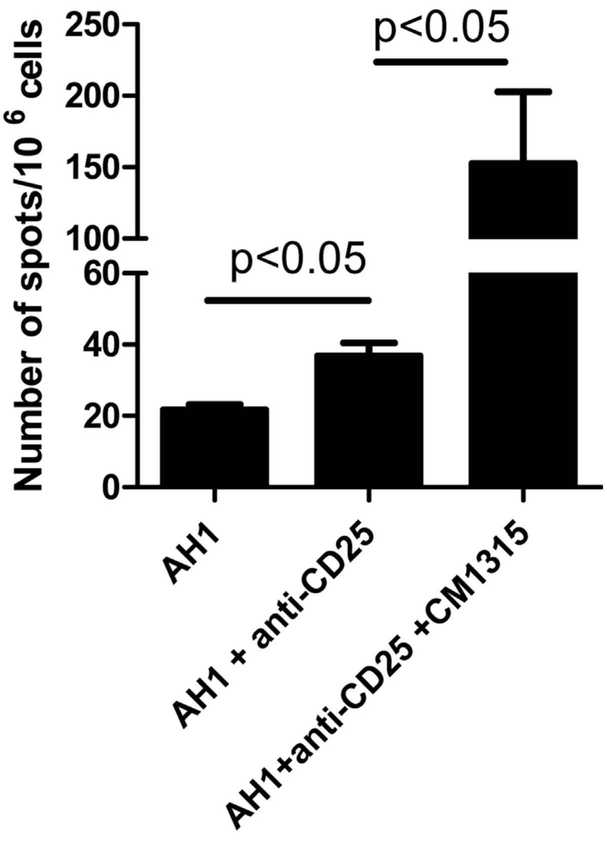

Supplementary Figure 6: *In vivo* the effect of CM-1315 peptide to improve the immunogenicity of AH1 peptide vaccine. AH1 peptide, encompassing a cytotoxic T cell epitope for H-2Ld MHC class I molecule, was emulsified in Incomplete Freund adjuvant as previously described [27] and used to immunize groups of naïve mice and in mice depleted of CD25+ T cells (n = 4). A group of CD25 depleted mice was immunized with AH1 and treated with CM-1315 peptide from day 1 to day 7 after immunization. Ten days after, immune response against AH1 was measured by ELISPOT assay to quantify the number of IFN-γ producing cells.

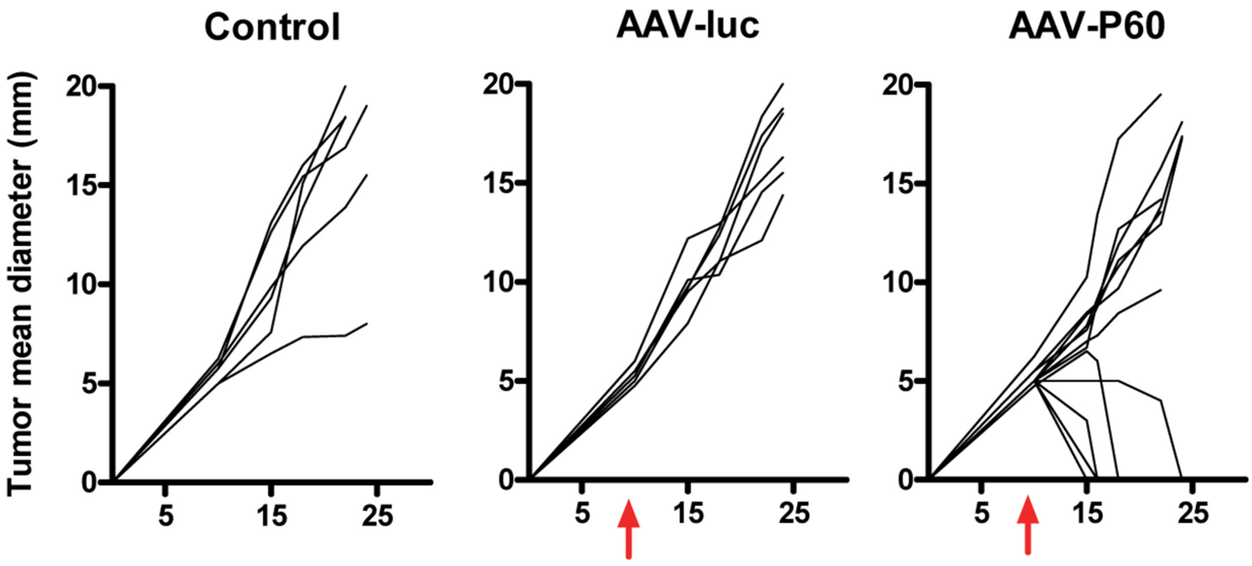

Supplementary Figure 7: Adeno-associated viruses (AAVs) were constructed with a transgene cassette encoding the reporter gene luciferase (AAV-Luc) or a minigene coding for the P60 peptide linked to a leader peptide (AAV-P60), under the transcriptional control of a hepatocyte-specific promoter (α1-antitrypsin promoter) as previously described [1]. Mice were challenged with Hepa129 tumor cells s.c and at day 10, when tumors reached 5 mm in diameter, they were treated i.t with 10^9 viral genome copies/mouse. Each curve represents tumor mean diameter for an individual mouse.

REFERENCES

1. Gil-Farina I, Di Scala M, Vanrell L, Olague C, Vales A, High KA, Prieto J, Mingozzi F, Gonzalez-Aseguinolaza G. IL12-mediated liver inflammation reduces the formation of AAV transcriptionally active forms but has no effect over preexisting AAV transgene expression. PLoS One. 2013; 8:e67748.
